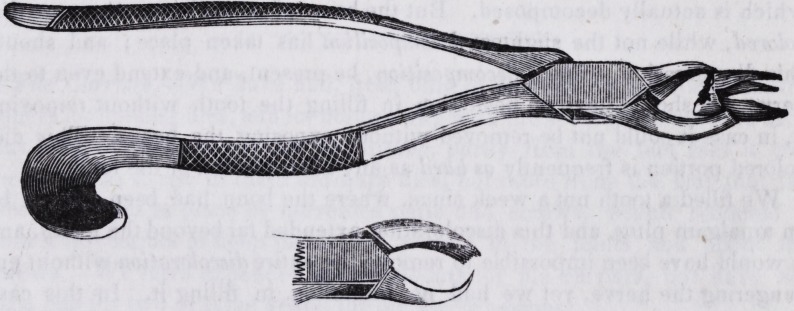# Forceps for Extracting Upper Molar Roots, When Not Separated

**Published:** 1847-03

**Authors:** 


*Forceps for Extracting Upper Molar Roots when not separated.*—The profession are now very well supplied with forceps for almost every variety of case which can present itself in practice. We do not recollect to have been put more to our wits-end in any cases of extraction than in removing upper molar roots, which are firm and not separated, and it has constituted about the only case where we have resorted to the turnkey for several years. When we used this instrument, we employed a *sharp hook,* and after removing the socket on the outside of the tooth, we placed the point of the hook in the *bifurcation* of the roots, and turned the tooth inwards. This mode of removing them would answer very well but for injuring the gum, which in such cases as require this method of operating, is generally in no condition to sustain pressure.

Some six or eight months since we altered over a common pair of upper molar forceps, by making the outer blade of the beak into a *hook,* and by pressing this between the roots, we could use the palatine root as a fulcrum, and thus save the pressure upon the soft parts which was inevitable with the turnkey.

On speaking of this method to Doctor Maynard, a few weeks since, he opened his case and produced two complete sets which he had used eight years! One pair had a hook upon the side as above described, and in the other a *blade* was substituted for the hook for *separating* the roots when it was difficult to extract them without. We procured one set of Dr. M. as a pattern, and had Mr. Arnold manufacture a set for ourself, only changing the form of the handles. Mr. A. informed me a few days since that several other pairs had been ordered. We regard it a great improvement over any other instrument in use for the purpose to which it is applied. The following is a drawing of the instrument, having a hook for the outer blade of the beak, as applied to a superior molar root.—*Syracuse Ed.*

## Figures and Tables

**Figure f1:**